# Non-invasive treatment in the management of Peyronie’s disease

**DOI:** 10.1177/1756287218823671

**Published:** 2019-02-11

**Authors:** Karen Randhawa, C. J. Shukla

**Affiliations:** Western General Hospital, Edinburgh, UK; Western General Hospital, Crewe Road South, Edinburgh, EH4 2XU, UK

**Keywords:** Peyronie’s disease, collagenase, intralesional injections, mechanical penile therapies, stem cell therapy

## Abstract

Peyronies disease (PD) is estimated to affect approximately 3–9% of men worldwide and maybe associated with pain, erectile dysfunction and penile deformity including shortening. The condition has significant debilitating effects on quality of life, self-esteem and psychological wellbeing in addition to sexual function. Surgical results add further to this by patients having dissatisfaction with various aspects of outcomes. Non-surgical management may allow patients to avoid the morbidities associated with surgery and still achieve improved functional and aesthetic outcomes. Several non-surgical options are currently being employed in the treatment of PD that may reduce or stabilize both objective measures (e.g. penile length and deformity) and subjective measures (including sexual function, pain and partner satisfaction). Nonsurgical management can allow patients to avoid the morbidities associated with surgery and still achieve improved functional and aesthetic outcomes. In this article we explore the current non-surgical management options for PD including oral, mechanical therapies, intralesional and topical treatments. We also briefly discuss future treatment options in the form of stem cell therapy.

## Introduction

Peyronie’s disease (PD) is characterized by a fibrous, inelastic lesion of the *tunica albuginea*. It is estimated to affect approximately 3–9% of men worldwide, although this figure may be higher in high-risk subgroups, for example, diabetes.^1–3^ The disease maybe associated with pain, erectile dysfunction, and penile deformity, including shortening. The condition has significant debilitating effects on quality of life, self-esteem and psychological wellbeing in addition to sexual function.^4^

Approximately 48% of men with PD suffer from depression (26% moderate, 22% severe) with 81% reporting emotional distress related to PD.^5,6^ These psychological effects are mostly due to changes in physical appearance and self-image induced by penile deformity. The resultant effects include reducing the quality and frequency of sexual relationships, reducing libido and intimacy, and resultant social and personal difficulties for relationships.^5,7,8^ A qualitative study of the psychosocial outcomes for patients with PD identified four core domains important to men including physical appearance and self-image, sexual function and performance, PD-related pain and discomfort and social stigmatization and isolation.^8^ Other themes highlighted include loss of sexual confidence and feelings of attractiveness, performance anxiety and concern about not satisfying partners sexually.^8^

Relationship stress was also reported with 54% of men reporting that PD had negatively impacted their relationship, demonstrating that the burden of the disease is shared by both patients and their partners.^9–11^ Partners of men with PD were found to have decreased sexual function, sexual satisfaction, and mood compared with population-based norms.^12^ Preliminary research has also shown negative partner reactions to be associated with lower sexual and relationship satisfaction in men with PD.^13^ Assessment of the emotional, psychosexual, and relationship aspects of the patient’s PD symptoms that negatively impact the patient’s quality of life, are needed to prioritize treatment goals.^14^

Despite multiple treatment options, PD remains a therapeutic dilemma due to an incomplete understanding of its aetiology, and the relative paucity of larger multi-institutional series and randomized, placebo-controlled trials.^15^ The pathophysiology is multifactorial in origin and is believed to occur as a result of aberrant penile wound healing in genetically susceptible individuals, who experience a localized response to endogenous factors [transforming growth factor beta (TGF-β)] released in response to microtrauma. This inflammatory response leads to the formation of fibrous inelastic plaque(s) within the bilayer of the *tunica albuginea*.^16,17^ The exact aetiology is unknown. Nonsurgical treatment modalities to date have focused on disrupting these processes.

Surgical options are suitable in correcting the deformity and aiding the ability to resume sexual function. However, they leave the patient with dissatisfaction due to loss of length of the phallus, residual deformity or erectile dysfunction necessitating the use of treatment.^18–20^ Evidence suggests that more than 65% of patients experience dissatisfaction following Lue’s procedures for PD.^21^ Additionally, satisfaction following penile prosthesis surgery for PD is lower than with the general population receiving the implant for other reasons, for example, vasculogenic or diabetic erectile dysfunction.^20^ The psychological effects of available surgical treatments and possible complications should all be taken into account when counselling patients regarding treatment options. Several nonsurgical options are currently being employed in the treatment of PD that may reduce or stabilize both objective measures (e.g. penile length and deformity) and subjective measures (including sexual function, pain and partner satisfaction).^22^ Nonsurgical management can allow patients to avoid the morbidities associated with surgery and still achieve improved functional and aesthetic outcomes.

## Course of Peyronie’s disease

There are two distinct phases of the disease; with the acute or active phase being characterized by active inflammation, penile pain and evolving deformity of the erect penis.^23^ The pain usually resolves by 12–18 months in 90% of men. After this phase, the vast majority of patients will progress on to stabilization of the plaque or progression of their disease. Approximately 3–13% of men describe spontaneous improvement,^24^ however, 30–50% of men will have progressive deterioration. The optimal time for nonsurgical intervention is likely during the active phase, when the inflammation is treatable.^25^ The chronic phase is characterized by stable curvature, pain resolution and the palpation of a hard, palpable plaque, although a plaque may not be present in all patients. Fibrosis, dystrophic calcifications and rarely ossification may occur in this phase.^26^

## Management options

Management options include mechanical, oral, intralesional and topical therapies. The nonsurgical management options with the most compelling contemporaneous evidence to support their use in PD are intralesional collagenase *Clostridium histolyticum* (CCH) and mechanical penile therapies, which may be used individually or in conjunction with one another.

### Intralesional CCH

CCH consists of purified enzymes (Auxilium [AUX] I and II) produced by the bacterium *C. histolyticum*. The collagen types I and III targeted by CCH are the most abundant fibres found in the plaques of Peyronie’s patients. When CCH is administered intralesionally, it cleaves type I and III collagens in a synergistic manner.^27^ CCH has also been shown to directly induce apoptosis in fibroblasts and decrease the expression of smooth muscle actin, transforming growth factor-β1 and fibronectin.^28^

CCH is the only drug approved by the US Food and Drug Administration (FDA) in 2013 for treatment of PD in men with dorsal or lateral penile curvature greater than 30° with or without a noncalcified penile plaque.^27^ It has also been approved in 2014 by the European Medicines Agency.

Initial studies have demonstrated its safety in randomized, placebo-controlled double-blind phase IIa trials which have also shown statistically significant improvement in penile curvature at an optimal dosage of 0.58mg.^29,30^ Subsequent studies have also tested the therapeutic effects seen with different treatment regimes with and without the use of modelling.^31^

Two large randomized double-blinded, placebo-controlled phase III trials in the US and Australia (IMPRESS I and II) have confirmed its efficacy. Each participant underwent up to four treatment cycles (consisting of two injections of either CCH 0.58 mg or placebo) 6 weeks apart, with approximately 24–72 h between injections. After the second injection of each cycle, patients underwent penile plaque modelling performed by the investigator, after which they were then instructed to perform home penile modelling three times per day during the 6-week period in between each treatment cycle.^32^

Patients treated with CCH showed a statistically significant improvement in penile curvature compared with those receiving placebo (34% *versus* 18.2% respectively) and significant improvement in Peyronie’s disease questionnaire (PDQ) bother score compared with placebo. Changes in penile plaque consistency were reported, in addition to improvement noted on International Index of Erectile Function (IIEF) overall satisfaction and PDQ psychological symptom score. These results have also been replicated in other phase III trials.^33^ Most common treatment-related adverse events (AEs) observed were mild to moderate in severity and were mostly localized to the injection area. The most frequently reported AEs (45% or greater) in CCH-treated men included penile ecchymosis, penile swelling and penile pain.^32^ Six patients experienced treatment-related serious AEs including three corporeal ruptures and three penile haematomas. All three corporeal ruptures and one penile haematoma required surgical repair, although the incidence of this may be higher and may not require surgical intervention based on a survey by the Sexual Medical Society of North America (SMSNA).^32,34^ The senior author CJS himself does not use any compressive dressing, and any bruising/oedema, etc. usually resolves by the end of 1 week following treatment and is mild enough to target a second injection into the plaque. The SMSNA Survey^34^ found that 37% of responders also recommended no dressing following injection. Interestingly, this survey also showed no increase of haematoma incidence was detected in patients in whom penile dressing was not applied and in anticoagulant- or platelet-using patients.

The International Consultation on Sexual Medicine (ICSM) guidelines^15^ recommend that the use of collagenase should be restricted to those with a stable curvature greater than 30° and less than 90°, no isolated hourglass deformity, calcified plaque or proximally located plaque at the base of the penis, with normal erectile function (grade B, level 2). As clinical trials have not evaluated the use of collagenase in patients with ventral curvature, calcified plaques, hourglass deformity or plaque located proximal to the base of the penis, it is uncertain as to whether these effects could be replicated in all PD patients.

The use of collagenase within the acute phase is also currently being assessed in trials where a mean curvature decrease of 20° after treatment was seen compared with 13.9° curvature seen in patients with chronic disease.^35^ There are limitations to this analysis based on the small number of patients in this trial, however early intervention during the acute phase could decrease penile deformity, the degree of penile fibrosis and subsequently decrease pain and sexual dysfunction.^36^

To date, clinical trials have not evaluated CCH use in ventral plaques, due to previous concerns of urethral injury during penile modelling.^37^

## Mechanical penile therapies

### Penile traction therapy (PTT)

The role of traction therapy in PD is to stop the progression of scarring, recover penile length and girth, reduce curvature, enhance sexual function and ultimately to avoid or simplify surgery.^38^

Application of mechanical stress modulates cell function through mechanotransduction, a cellular process that translates mechanical stimuli into a chemical response leading to activation of cell proliferation. Traction devices decrease myofibroblast activity and lead to upregulation of matrix metalloproteinases.^39^

European Association of Urology (EAU) guidelines state that ‘PTT may reduce penile deformity and increase penile length.’^40^ Preliminary studies have revealed that its regular use maybe associated with reduction in penile curvature, increase in flaccid penile length and improvement in penile pain.^41,42^

A recent nonrandomized, controlled trial assessed the use of penile traction in the acute phase of PD, assessing not only the impact on curvature, length and sexual function, but also correlation with sonographic penile evaluation. This trial showed a mean improvement in penile curvature of 20°, decreased pain and improved sexual function, in addition to disappearance of sonographic plaques in 48% of patients. The need for surgery was reduced in 40% of patients who would otherwise have been candidates for surgery. Predictors of success were penile curvature < 45% at baseline, time from diagnosis < 3 months, absence of plaque on the ultrasound study, age < 45 years and Visual Analogue Scale score for penile pain > 5.^43^

This study recommended the duration of PTT should not be shorter than 6 months, and must be worn at least 6 h a day to obtain these results. The therapy therefore requires a committed and compliant patient who is willing to devote time to a relatively long treatment period. PTT is, however, a tolerable minimally invasive method for men with PD.^44^

Studies are currently in process to help design an optimal protocol, assess efficacy of the different devices available and which men are most likely to benefit from their use. Further prospective, randomized, controlled studies with a larger number of patients and longer follow-up periods are needed. The ICSM guidelines support this, stating there may be some benefit in its use in PD patients (grade C, level 3).

Furthermore, the use of PTT concomitantly with either verapamil or interferon (IFN) α2b has also been shown an effective therapy.^45^ A study performed investigating the benefit of PTT with intralesional verapamil, oral L-arginine and pentoxifylline showed better curvature improvement and stretched penile length gain seen in the combination group. Additionally, length improvement was related to the duration of traction device use.

Daily PTT was assessed in men with PD who also underwent IFN α2b, however this study showed that PTT did not change penile girth. An important finding was that the use of PTT for 3 h or more resulted in a significant increase in stretched penile length of 0.31 cm compared with the use of PTT for less than 3 h.^46^

#### Combined use of PTT with collagenase *Clostridium histolyticum*

One study has been performed to evaluate the impact of combined use of PTT with CCH.^47^ Utilization patterns, attrition, and compliance issues were noted to be relevant factors impacting efficacy. PTT use declined in both frequency and duration with subsequent injection series. Overall, men treated with CCH exhibited significant decreases in penile curvature, consistent with the phase III trials (IMPRESS). When stratified by PTT, no statistically significant differences were identified in mean penile curvature improvement or stretched penile length, although only a minority of patients in the PTT arm were actually compliant with the therapy.

Further studies are currently being carried out to assess the optimal protocol; with a recently published study adopting three intralesional injections of CCH (0.9 mg) given at 4-weekly intervals using a new modified injection technique in an attempt to reduce cost and number of patient visits. A sum of 95.5 of patients in this study had an improvement in curvature with a mean value of 17.08° (0–40°) or 30.8% from baseline (0–57%) after three injections.^48^ One retrospective study has also assessed the relation between the number of treatment cycles and clinical outcomes for patients treated with CCH. Penile curvature was found to improve significantly after the first three treatment cycles but not the fourth, suggesting that further trials with fewer cycles may need further consideration. This trial also showed that patients who have a strong response to the first cycle of CCH are more likely to have a superior final decrease in penile curvature after completion of treatment cycles.^49^

A recent study has evaluated patient and partner satisfaction following CCH, showing 67% partner and 71% female sexual partner satisfaction with treatment respectively and is correlated with recall of prior penile trauma, improved ability to have sexual intercourse, and absence of post-procedural glans hypoesthesia.^50^ Goldstein and colleagues similarly had the same findings in their study based on 30 female partners of patients who had undergone CCH therapy.^51^

### Vacuum erection device

The role of vacuum erection device (VED) as a treatment for PD is less well established than PTT. Basic science studies suggest that VED results in dilation of cavernous sinuses, retrograde venous blood flow, and increased arterial inflow.^52,53^ The additional penile blood supply enhances cavernosal tissue oxygenation that subsequently leads to decreases in hypoxia-inducible factor-1a, TGF-β1, collagenase. This leads to increases in endothelial nitric oxide synthase and α-smooth muscle actin.^54^ When comparing the impact of VED and PTT in a rat model of PD, results showed enhanced preservation of α-smooth muscle actin and decreased TGF-β1 with VED *versus* PTT. The underlying mechanism could be related to antiapoptosis, antifibrosis, and smooth muscle preservation.^55^ There is, however, limited evidence establishing the effect of VED in different phases of PD.

The clinical evidence for its efficacy is based on one single-arm observational study by Raheem and colleagues on 31 patients using the device for 20 min per day.^56^ They noted that two thirds of their patients had a modest improvement of between 5–25°, with 10% having a worsening of their curvature and 23% experiencing no change. Their primary endpoint was a change in curvature, but not how many patients avoided surgery from this intervention.

Consensus statements on VED from the ICSM in 2015 suggested that, with the limited data available in PD, it could have a role as a primary treatment or postoperatively after incision or excision and grafting surgery.^15^

#### VED combined with CCH

Raheem and colleagues^48^ evaluated a shortened modified protocol of CCH in combination with VED showing an equal efficacy across all categories of curvatures as compared with the IMPRESS trials. This requires 4 instead of 14 visits, over 12 instead of 24 weeks. It remains to be seen in future trials whether VED is more efficacious with CCH as opposed to either treatment alone.

The remaining nonsurgical options can be divided by various delivery modalities into oral therapies, intralesional, and topical therapies.

## Oral therapies

Although oral treatments are an attractive option due to the ease of delivery, the evidence of outcomes from published series has proved disappointing. As a result, many international guidelines advise only limited use of these therapies based on the low level of evidence. The EAU guidelines state that oral treatment with potassium para-aminobenzoate may result in a significant reduction in penile plaque size and penile pain, as well as penile curvature stabilization with level 1b evidence.^40^

The published literature shows minimal or no benefit regarding the use of other oral therapies (e.g. vitamin E, colchicine, pentoxifylline) to significantly improve the penile deformity in PD; therefore, they are not recommended in the ICSM guidelines.^15^

## Intralesional injections

Intralesional injections, specifically IFN α2b, verapamil and CCH have proved more effective than topical and oral medications in the treatment of PD.

### Corticosteroids

Corticosteroids have been employed in PD due to their anti-inflammatory effects since the 1950s. Initial studies showed some objective improvement in penile curvature, however similar findings were also seen in the placebo groups.^57–62^ This raised the possibility that observed therapeutic effects seen with intralesional steroid injection were likely related to the mechanical effect of the injection rather than the drug on PD remodelling. Unfavourable side effects (wound infection, local tissue atrophy and fibrosis) coupled with limited efficacy do not support its use.

### Intralesional injection verapamil

The use of intralesional verapamil in PD was introduced in the mid 1990s, with initial studies showing an improvement in penile curvature and plaque volume using a biweekly series of injections, with several modifications to treatment regimens since.^63–67^

The rationale for intralesional use of verapamil (a calcium-channel antagonist) in patients with PD is based on *in vitro* research showing interference with fibroblast cellular proliferation.^68,69^ In animal models, histological evidence of cellular changes of decreased collagen and elastin fibres were seen, with improvement in penile pressures also noted.^70^

Intralesional verapamil has been evaluated in multiple randomized studies, including most recently, comparisons with tadalafil,^71^ hyaluronic acid,^72^ and thiocolchicine.^73^ In a trial comparing its use with tadalafil, intralesional verapamil did not improve plaque size or curvature degree with a mean baseline curvature of 20° in all groups with no statistically significant improvement in curvature.^73^ This was also confirmed in a prospective, double-arm, randomized, double-blinded study that showed no improvement in penile curvature with verapamil, although this concluded a greater efficacy of hyaluronic acid in terms of penile curvature and patient satisfaction in over 100 patients when compared with verapamil. However, the mean baseline curvature was 33°, with change following treatment deemed as between 0° and 4° decrease in curvature.^71^

Intralesional thiocolchicine, achieved similar results to verapamil, with improvement in curvature reported in 69% of cases treated with thiocolchicine and in 66% of those who received verapamil. However, the study was limited due to its small size (only 25 patients) and the improvement in curvature which was not deemed statistically significant with either treatment.^73^

Comparisons among previously published noncontrolled, single-arm, prospective clinical trials on intralesional verapamil injections have shown a greater decrease in penile curvature after prolonged treatment with 12 injections over 6 months compared with 6 injections over 3 months.^74,75^

It appears that the injected volume, frequency, concentration, and duration of the injection protocol affects outcome results. Longer treatment periods of concentrated intralesional verapamil in younger men with small plaques but large curvature have been shown to receive the optimal benefit.^76,77^

Overall, the evidence suggests that intralesional verapamil injections could be advocated for the treatment of noncalcified acute phase or chronic plaques to stabilise disease progression or reduce penile curvature, although no optimal treatment regime exists at present. The ICSM guidelines support that intralesional verapamil has shown some outcome benefits in PD management^15^ (grade C, level 3).

Common reported AEs include penile bruising, swelling, and pain at the injection site, however, dizziness, nausea, sweating and loss of libido have also been reported in rare cases.^78^

### Intralesional interferon α2b

IFN α2b has been shown to decrease extracellular matrix production, inhibit fibroblast proliferation, and therefore decrease collagen production from fibroblasts and improve the wound healing process from PD plaques *in vitro*.^79,80^ Evidence also suggests that IFN leads to an improvement in penile haemodynamics, thereby improving erectile function.^81,82^

A double-blind, placebo-controlled trial^80^ showed that intralesional IFN α2b, when injected biweekly for 12 weeks, led to a modest but significant improvement in penile curvature (13° *versus* 4° in the placebo arm), decreased pain relief and plaque size. A more recent study also supported these findings, demonstrating a statistically significant improvement in curvature with intralesional IFN α2b, without affecting vascular parameters.^83^ The absolute improvement in curvature was found to be independent of pretreatment curvature or duration of disease. Recent evidence has also shown this improvement to be independent of plaque location with a >20% reduction in curvature seen in the majority of men with PD when treated with intralesional IFN α2b.^84^

IFN α2b injections have a good safety profile, with the most common AEs being self-limiting flu-like symptoms.

The ICSM guidelines state that intralesional IFN has shown some outcome benefits in PD management^15^ (grade B, level 2).

### Hyaluronic Acid (HA)

Intralesional HA decreases inflammatory cytokines, and thereby reduces inflammation and scar formation. This is a relatively novel treatment that appears to have some efficacy in improving symptoms, however data comparing HA treatment with placebo or alternative therapies are lacking.

In one recent study, 81 patients in the active phase of the disease underwent a 10-week cycle of weekly plaque injections.^85^ HA demonstrated statistically significant improvement over controls in plaque size, penile curvature and improvement in penile rigidity at 12 months. Improvements remained stable at 24 months.

In a prospective, single-arm, multicentre pilot study, 65 patients underwent a 10-week cycle of weekly intralesional injections with HA. This study showed a significant decrease in plaque size, and decreased penile curvature in 37% with improvement in overall sexual satisfaction.^86^ These findings were also confirmed in a prospective, double-arm, randomized, double-blinded study showing greater efficacy of HA in terms of penile curvature and patient satisfaction compared with intralesional verapamil.^72^ Although plaque size and overall sexual satisfaction improved with both therapies, better outcomes were observed in patients treated with HA, although not deemed statistically significant. Further prospective, randomized controlled trials (RCTs) will need to be performed prior to bring about the routine recommendation of HA.

HA has a lower risk of adverse effects compared with other molecules used ‘off label’ for intralesional therapy. Its use is also being evaluated in electromotive administration comparing the effects with verapamil. However, although HA proved to be more efficient than verapamil in both reducing curvature, pain and plaque area, and in improving erectile function, this was not of statistical significance and was evaluated in patients with curvature < 30°.

### Plasma-rich platelets (PRP) + Hyaluronic Acid (HA)

PRP injections have been used to improve angiogenesis and wound healing, and could theoretically improve ED, PD and stress urinary incontinence.^88^

PRP is derived from the centrifugation of whole blood with a separator gel to remove the red and white blood cells. The resulting supernatant has a greater-than-fourfold increase in platelets and other plasma proteins.^89^ This concentrate is then administered *via* injection into the lesion combined with HA.

There have been limited trials into its use to date, mainly by Virag et al.^90^ In a recent trial, 90 patients were injected intralesionally under ultrasound guidance with four to eight sessions applied at 15-day intervals for the first four and monthly thereafter.^90^ Changes in PDQ, IIEF-5, angulation and maximum thickness were evaluated 1 month after the last session. Average angulations (curvature or deformation) were reduced by 39.65%; with the mean final improvement 16.54 ± 10.51°. The average maximum thickness diminished by 1.1 mm, with the average PDQ score decreased by 5.5. IIEF-5 improved significantly, with 43.3% judging that their erections were better and 46.7% perceiving that sexual activity was easier following the treatment. Superficial haematomas were observed in 10% of the injection sites with ecchymosis present in 16.7% of patients.

PRP with HA has been demonstrated efficiency in 70%, without any severe complication. If confirmed in additional and larger series, PRP with HA could be adopted as a cost-effective minimally invasive treatment of PD.

## Topical therapies

Topical verapamil is not recommended in Peyronie’s patients^15^ (ICSM guidelines grade B, level 3). These recommendations are based on a study where men who were pretreated with topical verapamil then immediately underwent surgical correction for their PD.^91^ These excised samples (mean 1.1 g) of tunica failed to show the presence of any verapamil, demonstrating that the gel does not infiltrate the *tunica albuginea*.

### Electromotive drug administration/Iontophoresis

Iontophoresis involves the transport of ions through tissue by means of an electric current, causing electrokinetic repulsion of positively charged medication toward the diseased target tissue, limiting systemic side effects.^92^ It is also thought to provide superior tissue penetration for the transdermal application of medications.^22^

Excised Peyronie’s plaques following electromotive drug therapy with dexamethasone, verapamil and lidocaine have been shown to have decreased expression of basic fibroblast growth factor.^93^ Despite initial trials showing promising results on plaque size, curvature and pain using a variety of different pharmacologic agents over the course of short, three-week treatment regimes, a subsequent RCT demonstrated that there was no superiority of the use of verapamil alone compared with saline placebo.^94–96^

A recent study compared the administration of combination therapy of verapamil and dexamethasone administered *via* electromotive drug administration (EMDA) with intralesional administration; although EMDA administration showed improvements with regards to plaque length, plaque width, penile curvature, plaque volume and erectile dysfunction, these were not statistically significant.

The ICSM guidelines have therefore not recommended the use of iontophoresis in PD patients (grade B, level 3). ^15^

### Topical H-100 gel

H-100 gel combines a natural carrier agent, emu oil, with nicardipine and superoxide dismutase. Emu oil has an anti-inflammatory effect, demonstrated in animal studies and, when applied topically, decreases the levels of pro-inflammatory cytokines in tissue and therefore inhibits local secondary inflammation to promote wound healing.^97,98^ In a double-blind, placebo-controlled pilot study designed to assess safety, 22 patients in the acute phase of PD were randomized to receive 3 months of either placebo or H-100 gel to be applied focused on the plaque, but to also cover the entire shaft. Following these 3 months, all study participants received H-100 gel for a further 3 months.^99^

In the treatment group, significant reductions in penile curvature and pain level were seen, as well as an increase in penile length. The drug was well tolerated overall, with a self-limiting skin rash as the only adverse event. Although this trial was designed to assess safety, its initial positive outcomes make it an encouraging novel treatment although more efficacy and safety data from larger trials are required prior to recommendation for usage.^100^

## Low-intensity extracorporeal shockwave therapy

Local penile low-intensity extracorporeal shockwave therapy (Li-ESWT) has been used to treat PD with mixed results. The hypotheses behind its mode of action include direct damage to the plaque, resulting in an inflammatory reaction with increased macrophage activity leading to plaque lysis, improved vascularity resulting in plaque resorption, and the creation of contralateral scarring of the penis resulting in ‘false’ straightening.^101^

Most uncontrolled studies have failed to show any significant improvements in patients with PD.^102–104^ Increasing evidence suggests that Li-ESWT has minimal impact on deformity correction but provides a more rapid decrease of pain and stabilization of curvature in patients with PD (grade B, level 3).^15,105,106^ The treatment may not be recommended, as pain may resolve spontaneously in the natural history of disease and the deviation may worsen with ESWT. Its potential side effects, including penile fibrosis and development of erectile dysfunction, have precluded its widespread use.^107,108^

Overall, the published literature has largely failed to demonstrate any significant benefit in treating penile curvature, however outcomes are interpreted with caution due to methodological flaws. There is anticipation of future studies clarifying ESWTs role in PD curvature improvement/resolution more clearly.

## Stem-cell therapy/Regenerative medicinal therapies

There is now increasing evidence for the role of mesenchymal stem cells (MSCs) as a potential treatment for fibrosis in PD. These cells originate from fetal tissue, umbilical cord blood, or adult tissues and may also exert beneficial effects promoting local growth, repair, and regeneration of the tissue in which they reside.^109–111^

Studies involving rat models have been performed to determine the effects of stem-cell treatment in PD.^112,113^ Injection of adipose tissue–derived stem cells (ADSC) into the tunica albuginea in rat models during the acute phase of PD prevented formation of fibrosis in the tunica and corpus cavernosum and statistically significantly improved erectile function.^112^ Another study demonstrated that ADSCs combined with IFN α2β injections prevented or reduced Peyronie’s plaques by decreasing the expression of tissue inhibitors of metalloproteinases.^113^ They showed that ADSCs, both alone and in combination with IFN, resulted in improved erectile response and decreased PD-like manifestations in a PD rat model.

To date, there have been very few studies performed in humans.^114^ In one prospective study, patients with PD were injected with PM-MSCs (placental-matrix-derived mesenchymal stem cells) and followed up to assess changes in plaque volume, penile curvature, and erectile function.^114^ Of a total of 10 plaques managed, 7 had disappeared completely at 3-month follow up. Changes in end-diastolic velocity, stretched penile length and penile girth were not statistically significant. Both penile curvature and peak systolic velocity were significantly improved at 6 weeks, 3 months, and 6 months after PM-MSC injection, but end diastolic volume (EDV) did not prove to demonstrate a statistically significant improvement. The results suggest that PM-MSCs may be beneficial and effective as a nonsurgical treatment in PD patients.

It seems evident in experimental settings that stem cells in general (ADSCs in particular) provide a feasible, safe and effective therapy for PD.^114^ However, further prospective human studies are needed to further elucidate the therapeutic potential of stem-cell therapy in PD.

## Conclusion

Oral therapies serve a very limited role in treatment due to their limited efficacy and are not currently recommended for use.^15^ Mechanical therapies and vacuum extender devices have been shown to provide some benefit, although they require daily use and a motivated patient to continue long-term use.

Intralesional CCH is the first FDA-approved medication and can be considered a reasonable alternative to surgery for patients desiring conservative treatment, based on its improvement in penile curvature and symptom bother. Alternative intralesional therapies are promising, however additional large studies are needed to evaluate their efficacy prior to recommendations regarding their use. Topical treatments (for example verapamil and iontophoresis) also have mixed results based on the published literature and are not currently recommended for use.^15^ Currently, it is likely, in the context of nonsurgical treatments, that a multimodal approach, for example, combination of mechanical therapies and CCH, is the most suitable way forward in managing this condition.

Although stem-cell therapy offers a promising potential future treatment, it is still in the experimental phase with paucity of data supporting its use and long-term effects at present.

Important advances in the physical treatment of penile curvature in PD do not however negate the need for the development and examination of effective educational, psychological, and psychosexual interventions to address unmet needs in PD.^115^
